# High-quality permanent draft genome sequence of *Rhizobium leguminosarum* bv. *viciae* strain GB30; an effective microsymbiont of *Pisum sativum* growing in Poland

**DOI:** 10.1186/s40793-015-0029-6

**Published:** 2015-07-16

**Authors:** Andrzej Mazur, Sofie E. De Meyer, Rui Tian, Jerzy Wielbo, Kamil Zebracki, Rekha Seshadri, TBK Reddy, Victor Markowitz, Natalia N. Ivanova, Amrita Pati, Tanja Woyke, Nikos C. Kyrpides, Wayne Reeve

**Affiliations:** Department of Genetics and Microbiology, Maria Curie Sklodowska University, Lublin, Poland; Centre for Rhizobium Studies, Murdoch University, Murdoch, Western Australia; DOE Joint Genome Institute, Walnut Creek, California USA; Biological Data Management and Technology Center, Lawrence Berkeley National Laboratory, Berkeley, California USA; Department of Biological Sciences, King Abdulaziz University, Jeddah, Saudi Arabia

**Keywords:** Root-nodule bacteria, Nitrogen fixation, Rhizobia, Alphaproteobacteria, GEBA-RNB

## Abstract

*Rhizobium leguminosarum* bv. *viciae* GB30 is an aerobic, motile, Gram-negative, non-spore-forming rod that can exist as a soil saprophyte or as a legume microsymbiont of *Pisum sativum*. GB30 was isolated in Poland from a nodule recovered from the roots of *Pisum sativum* growing at Janow. GB30 is also an effective microsymbiont of the annual forage legumes vetch and pea. Here we describe the features of *R. leguminosarum* bv. *viciae* strain GB30, together with sequence and annotation. The 7,468,464 bp high-quality permanent draft genome is arranged in 78 scaffolds of 78 contigs containing 7,227 protein-coding genes and 75 RNA-only encoding genes, and is part of the GEBA-RNB project proposal.

**Electronic supplementary material:**

The online version of this article (doi:10.1186/s40793-015-0029-6) contains supplementary material, which is available to authorized users.

## Introduction

The most efficient biological nitrogen fixation occurs when bacterial microsymbionts (rhizobia) form an effective symbiotic association with legume host plants. Legumes can develop these interactions with many different species of rhizobia belonging mainly to the *Alphaproteobacteria*, including *Azorhizobium*, *Allorhizobium**,**Bradyrhizobium**,**Ensifer**,**Mesorhizobium* and *Rhizobium* [1, 2]. The genus *Rhizobium* contains at the time of writing 71 species, and within a species there may be distinct symbiovars [3].

Within the species *Rhizobium leguminosarum*, there are three distinct symbiovars [4, 5] including bv. *phaseoli* that forms nodules with *Phaseolus vulgaris*, bv. *trifolii* that forms nodules with clover (*Trifolium*) and bv. *viciae* that forms nodules on vetch, pea and lentil (*Vicia**,**Lathyrus**, Pisum* and *Lens*). In *R. leguminosarum* the *nod* genes that define these distinct host specificities are mostly located on the symbiotic plasmid, which has generically been designated pSym. The genomes of *R. leguminosarum* strains are usually large and complex containing, in addition to pSym, a chromosomal replicon and extra-chromosomal low-copy-number replicons characterized by the presence of *repABC* replication systems [6–8]. Recent studies have revealed that substantial divergence can occur in this genome organization and in the metabolic versatility of *R. leguminosarum* isolates [5, 9–12]. Kumar *et al.* [5] demonstrated that the diversity of *R. leguminosarum* within a local population of nodule isolates was 10 times higher than that found for *Ensifer medicae*. It was noted that the abundance of a particular genotype within the population can vary significantly and adaptation to the edaphic environment is a sought after trait particularly for the development of inoculants [13, 14].

*R. leguminosarum* bv. *viciae* GB30 was isolated as the most abundant nodule inhabitant (>42 %) of *Pisum sativum* cv. Ramrod plants cultivated at a field site in Janow, Poland [10]. In contrast to other abundant isolates, GB30 formed nodules and fixed nitrogen with both *P. sativum* and *Vicia villosa* (cv. Wista). Preliminary investigation into the genome architecture using Eckhardt analysis has revealed that GB30 contained a multipartite genome consisting of six replicons with one chromosome and five plasmids [10]. The genome of this strain could therefore provide important insights into the mechanisms required by effective *R. leguminosarum* microsymbionts to adapt to a particular edaphic environment. Here, we present a set of general features for *Rhizobium leguminosarum* bv. *viciae* GB30 together with the description of the complete genome sequence and annotation.

## Organism information

### Classification and features

**Fig. 1:**
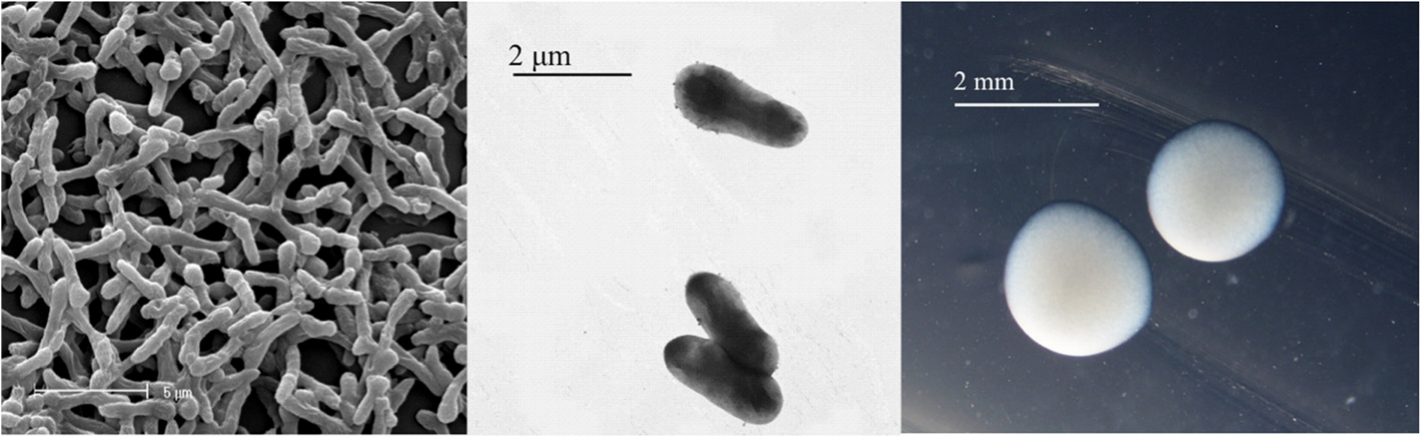
Images of *Rhizobium leguminosarum* bv. *viciae* strain GB30 using scanning (Left) and transmission (Center) electron microscopy and the appearance of colony morphology on ½LA solid media (Right)

*R. leguminosarum* bv. *viciae* strain GB30 is a motile, Gram-negative rod in the order *Rhizobiales* of the class *Alphaproteobacteria*. The rod-shaped form varies in size with dimensions of 0.8-1 μm in width and 2.3-2.5 μm in length (Fig. 1 Left and Center). It is fast growing, forming colonies within 3–4 days when grown on half strength Lupin Agar (½LA) [15] at 28 °C. Colonies on ½LA are white-opaque, slightly domed and moderately mucoid with smooth margins (Fig. 1 Right).

**Fig. 2:**
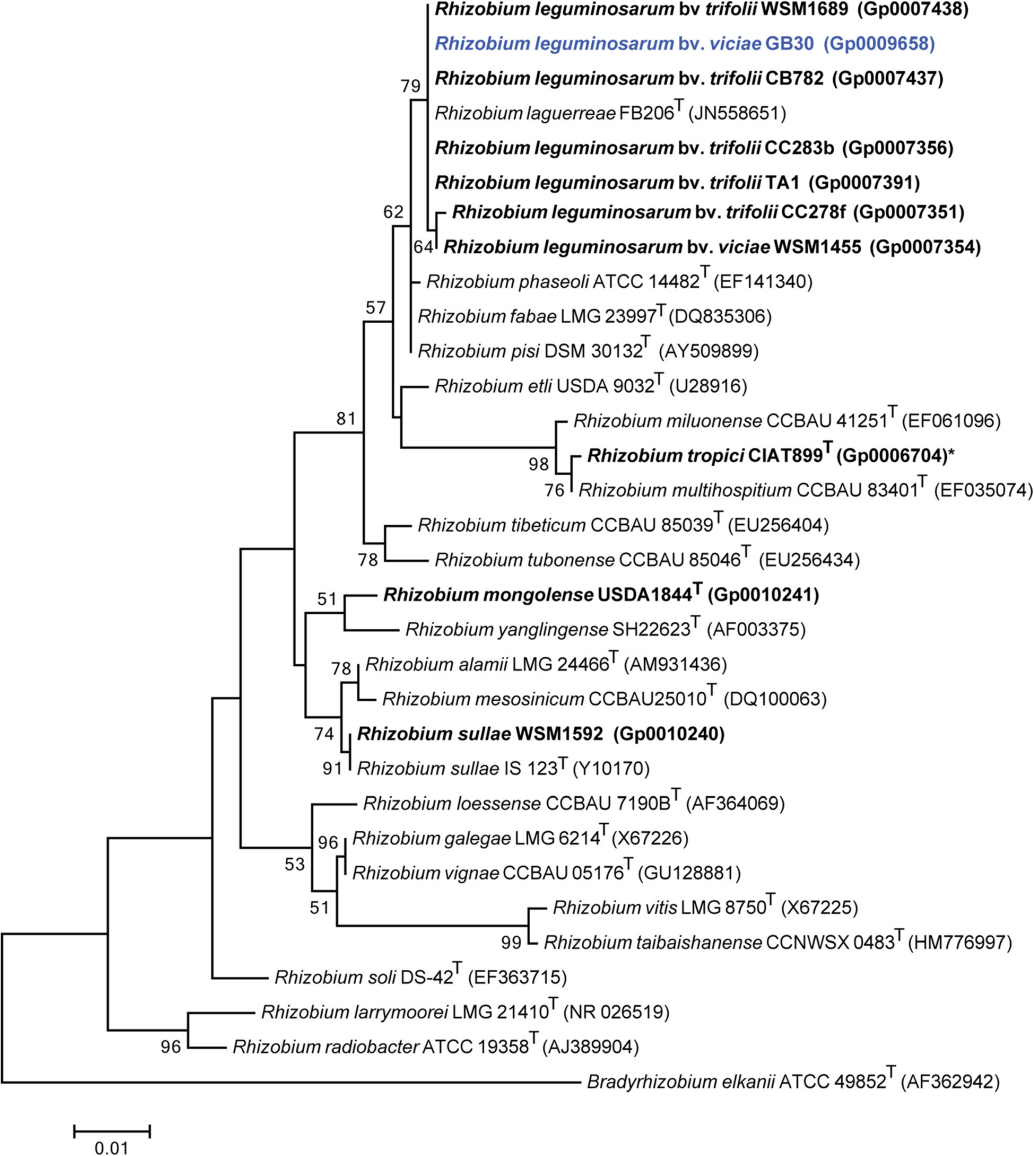
Phylogenetic tree highlighting the position of *Rhizobium leguminosarum* bv. *viciae* GB30 (shown in blue print) relative to other type and non-type strains in the *Rhizobium* genus using a 901 bp internal region of the 16S rRNA gene. *Bradyrhizobium elkanii* ATCC 49852^T^ was used as outgroup. All sites were informative and there were no gap-containing sites. Phylogenetic analyses were performed using MEGA, version 5.05 [36]. The tree was built using the maximum likelihood method with the General Time Reversible model. Bootstrap analysis with 500 replicates was performed to assess the support of the clusters. Type strains are indicated with a superscript T. Strains with a genome sequencing project registered in GOLD [20] are shown in bold and have the GOLD ID mentioned after the strain number, otherwise the NCBI accession number has been provided. Finished genomes are designated with an asterisk

Figure 2 shows the phylogenetic relationship of *Rhizobium leguminosarum* bv. *viciae* GB30 in a 16S rRNA gene sequence based tree. This strain is phylogenetically most related to *Rhizobium laguerreae* FB206^T^ and *Rhizobium gallicum* R602sp^T^ based on the 16S rRNA gene alignment with sequence identities of 100 %, as determined using the EzTaxon-e server [16]. *Rhizobium laguerreae* FB206^T^ was isolated from effective *Vicia faba* root nodules in Tunisia [17], whereas *Rhizobium gallicum* R602sp^T^ was isolated from effective *Phaseolus vulgaris* root nodules in France [18]. Sequence similarity was also investigated with strains from the GEBA-RNB project [12] and GB30 was found to be closely related to *R. leguminosarum* bv. *trifolii* WSM1689 with 100 % 16S rRNA gene sequence identity. *R. leguminosarum* bv. *trifolii* WSM1689 is a highly effective microsymbiont of the perennial clover *Trifolium uniflorum* and has been shown to have a remarkable narrow host range [19]. Minimum Information about the Genome Sequence (MIGS) is provided in Table 1 and Additional file 1: Table S1.

**Table 1:** Classification and general features of Rhizobium leguminosarum bv. viciae strain GB30 in accordance with the MIGS recommendations [37] published by the Genome Standards Consortium [38].

MIGS ID	Property	Term	Evidence code
		Domain *Bacteria*	TAS [39]
		Phylum *Proteobacteria*	TAS [40, 41]
		Class *Alphaproteobacteria*	TAS [42, 43]
	Classification	Order *Rhizobiales*	TAS [44]
		Family *Rhizobiaceae*	TAS [45]
		Genus *Rhizobium*	TAS [46]
		Species *Rhizobium leguminosarum*	TAS [47–49]
	Gram stain	Negative	IDA
	Cell shape	Rod	IDA
	Motility	Motile	IDA
	Sporulation	Non-sporulating	NAS
	Temperature range	Mesophile	NAS
	Optimum temperature	28 °C	TAS [9]
	pH range; Optimum	Not reported	
	Carbon source	Not reported	
MIGS-6	Habitat	Soil, root nodule, on host	TAS [9]
MIGS-6.3	Salinity	Non-halophile	NAS
MIGS-22	Oxygen requirement	Aerobic	TAS [49]
MIGS-15	Biotic relationship	Free living, symbiotic	TAS [10]
MIGS-14	Pathogenicity	Non-pathogenic	TAS [50]
MIGS-4	Geographic location	Janow, near Lublin, eastern Poland	TAS [10]
MIGS-5	Sample collection	Between May and June, 2008	TAS [10]
MIGS-4.1	Latitude	51.387638	TAS [10]
MIGS-4.2	Longitude	22.369194	TAS [10]
MIGS-4.3	Altitude	185 m	IDA

Evidence codes – IDA: Inferred from Direct Assay; TAS: Traceable Author Statement (i.e., a direct report exists in the literature); NAS: Non-traceable Author Statement (i.e., not directly observed for the living, isolated sample, but based on a generally accepted property for the species, or anecdotal evidence). These evidence codes are from the Gene Ontology project [51].

#### Symbiotaxonomy

*R. leguminosarum* bv. *viciae* strain GB30 was obtained from pea nodules (*P. sativum* cv. Ramrod) growing in sandy loam (N:P:K 0.157:0.014:0.013 %) in Janow near Lublin (Poland). The soil contained a relatively high number of *R. leguminosarum* bv. *viciae*, bv. *trifolii* and bv. *phaseoli* cells i.e., 9.2 × 10^3^, 4.2 ÷ 10^3^ and 1.5 × 10^3^ bacteria/g of soil, respectively, as determined by the most probable number (MPN) method [10]. Plants were grown on 1 m^2^ plot for six weeks between May and June, 2008. Five randomly chosen pea plants growing in each other’s vicinity were harvested; the nodules were collected, surface-sterilized and the microsymbionts isolated [10]. One of the most abundant isolates, GB30, formed nodules (Nod^+^) and fixed N_2_ (Fix^+^) with *P. sativum* and *Vicia villosa* (cv. Wista) increasing the wet mass weight by 54 and 38 %, respectively. Plants inoculated with GB30 also showed a 2.6 fold increase in nodule number and a 2.2 fold increase in seed pod number.

## Genome sequencing and annotation information

### Genome project history

This organism was selected for sequencing on the basis of its environmental and agricultural relevance to issues in global carbon cycling, alternative energy production, and biogeochemical importance, and is part of the Genomic Encyclopedia of Bacteria and Archaea, The Root Nodulating Bacteria chapter (GEBA-RNB) project at the U.S. Department of Energy, Joint Genome Institute [12]. The genome project is deposited in the Genomes OnLine Database [20] and the high-quality permanent draft genome sequence in IMG [21]. Sequencing, finishing and annotation were performed by the JGI using state of the art sequencing technology [22]. A summary of the project information is shown in Table 2.

**Table 2:** Genome sequencing project information for Rhizobium leguminosarum bv. viciae strain GB30

MIGS ID	Property	Term
MIGS-31	Finishing quality	High-quality permanent draft
MIGS-28	Libraries used	Illumina Std PE
MIGS-29	Sequencing platforms	Illumina Hiseq 2000
MIGS-31.2	Fold coverage	121.9 x Illumina
MIGS-30	Assemblers	Velvet version 1.1.04; ALLPATHS v. r41043
MIGS-32	Gene calling methods	Prodigal 1.4
	Locus Tag	A3A3
	GenBank ID	ATTP00000000
	GenBank Date of Release	July 9, 2013
	GOLD ID	Gp0009658 [52]
	BIOPROJECT	PRJNA165299
MIGS-13	Source Material Identifier	GB30
	Project relevance	Symbiotic N_2_ fixation, agriculture

### Growth conditions and genomic DNA preparation

*R. leguminosarum* bv. *viciae* strain GB30 was grown to mid logarithmic phase in TY rich media [23] on a gyratory shaker at 28 °C. DNA was isolated from 60 mL of cells using a CTAB (Cetyl trimethyl ammonium bromide) bacterial genomic DNA isolation method [24].

### Genome sequencing and assembly

The draft genome of *Rhizobium leguminosarum* bv. *viciae* GB30 was generated at the DOE Joint Genome Institute [22]. An Illumina Std shotgun library was constructed and sequenced using the Illumina HiSeq 2000 platform which generated 25,943,396 reads totaling 3,891.5 Mbp. All general aspects of library construction and sequencing performed at the JGI can be found at the JGI web site [25]. All raw Illumina sequence data was passed through DUK, a filtering program developed at JGI, which removes known Illumina sequencing and library preparation artefacts (Mingkun L, Copeland A, Han J. unpublished). Following steps were then performed for assembly: (1) filtered Illumina reads were assembled using Velvet version 1.1.04 [26] (2) 1–3 Kbp simulated paired end reads were created from Velvet contigs using wgsim [27] (3) Illumina reads were assembled with simulated read pairs using Allpaths–LG (version r41043) [28]. Parameters for assembly steps were: 1) Velvet (velveth: 63 –shortPaired and velvetg: −very_clean yes –export-Filtered yes –min_contig_lgth 500 –scaffolding no –cov_cutoff 10) 2) wgsim (−e 0 –1 100 –2 100 –r 0 –R 0 –X 0) 3) Allpaths–LG (PrepareAllpathsInputs: PHRED_64 = 1 PLOIDY = 1 FRAG_COVERAGE = 125 JUMP_COVERAGE = 25 LONG_JUMP_COV = 50, RunAllpathsLG: THREADS = 8 RUN = std_shredpairs TARGETS = standard VAPI_WARN_ONLY = True OVERWRITE = True). The final draft assembly contained 78 contigs in 78 scaffolds. The total size of the genome is 7.5 Mbp and the final assembly is based on 910.4 Mbp of Illumina data, which provides an average of 121.9× coverage.

### Genome annotation

Genes were identified using Prodigal [29], as part of the DOE-JGI genome annotation pipeline [30, 31]. The predicted CDSs were translated and used to search the National Centre for Biotechnology Information (NCBI) non-redundant database, UniProt, TIGRFam, Pfam, KEGG, COG, and InterPro databases. The tRNAScanSE tool [32] was used to find tRNA genes, whereas ribosomal RNA genes were found by searches against models of the ribosomal RNA genes built from SILVA [33]. Other non–coding RNAs such as the RNA components of the protein secretion complex and the RNase P were identified by searching the genome for the corresponding Rfam profiles using INFERNAL [34]. Additional gene prediction analysis and manual functional annotation was performed within the Integrated Microbial Genomes-Expert Review (IMG-ER) system [35] developed by the Joint Genome Institute, Walnut Creek, CA, USA.

## Genome Properties

The genome is 7,468,464 nucleotides with 60.81 % GC content (Table 3) and comprised of 78 scaffolds of 78 contigs. From a total of 7,302 genes, 7,227 were protein encoding and 75 RNA only encoding genes. The majority of genes (79.57 %) were assigned a putative function whilst the remaining genes were annotated as hypothetical. The distribution of genes into COGs functional categories is presented in Table 4.

**Table 3:** Genome Statistics for Rhizobium leguminosarum bv. viciae strain GB30

Attribute	Value	% of Total
Genome size (bp)	7,468,464	100.00
DNA coding (bp)	6,497,898	87.00
DNA G + C (bp)	4,541,558	60.81
DNA scaffolds	78	100.00
Total genes	7,302	100.00
Protein coding genes	7,227	98.97
RNA genes	75	1.03
Pseudo genes	0	0
Genes in internal clusters	470	6.44
Genes with function prediction	5,810	79.57
Genes assigned to COGs	5,182	70.97
Genes with Pfam domains	6,025	82.51
Genes with signal peptides	634	8.68
Genes with transmembrane proteins	1,646	22.54
CRISPR repeats	1

**Table 4:** Number of genes associated with the general COG functional categories.

Code	Value	% age	Description
J	233	3.90	Translation, ribosomal structure and biogenesis
A	0	0.00	RNA processing and modification
K	597	9.98	Transcription
L	128	2.14	Replication, recombination and repair
B	2	0.03	Chromatin structure and dynamics
D	35	0.59	Cell cycle control, Cell division, chromosome partitioning
V	119	1.99	Defense mechanisms
T	285	4.77	Signal transduction mechanisms
M	310	5.18	Cell wall/membrane/envelope biogenesis
N	93	1.56	Cell motility
U	58	0.97	Intracellular trafficking, secretion, and vesicular transport
O	206	3.44	Posttranslational modification, protein turnover, chaperones
C	325	5.43	Energy production and conversion
G	644	10.77	Carbohydrate transport and metabolism
E	689	11.52	Amino acid transport and metabolism
F	116	1.94	Nucleotide transport and metabolism
H	270	4.52	Coenzyme transport and metabolism
I	241	4.03	Lipid transport and metabolism
P	317	5.30	Inorganic ion transport and metabolism
Q	186	3.11	Secondary metabolite biosynthesis, transport and catabolism
R	695	11.62	General function prediction only
S	381	6.37	Function unknown
-	2,120	29.03	Not in COGS

The total is based on the total number of protein coding genes in the genome.

## Conclusion

*Rhizobium leguminosarum* bv. *viciae* GB30 belongs to a group of Alpha-rhizobia strains isolated from *Pisum sativum* in Poland. Strain GB30 is part of the GEBA-RNB project that sequenced 24 *R. leguminosarum* strains and 12 *R. leguminosarum* bv. *viciae* strains [12]. Phylogenetic analysis revealed that GB30 is most closely related to *Rhizobium leguminosarum* bv. *trifolii* CB782 and WSM1689, both part of the GEBA-RNB project [12]*.* Full genome comparison of GB30 and WSM1689 [19] revealed that GB30 has the largest genome (7.4 Mbp), with the highest COG count (5,182), the lowest Pfam % (82.51) and the lowest TIGRfam % (22.13 %). The genome attributes of *R. leguminosarum* bv. *viciae* GB30, in conjunction with the other *R. leguminosarum* genomes, will be important for on-going comparative and functional analyses of the plant microbe interactions required for the successful establishment of agricultural crops.

## Additional file

Additional file 1: Table S1. Associated MIGS record.

## Abbreviations

GEBA-RNB: Genomic Encyclopedia of Bacteria and Archaea – Root Nodule Bacteria
JGI: Joint Genome Institute
TY: Trypton Yeast
CTAB: Cetyl trimethyl ammonium bromide
WSM: Western Australian Soil Microbiology
MPN: Most probable number
IMG-ER: Integrated Microbial Genomes-Expert Review
NCBI: National Centre for Biotechnology Information
