# Supplementary material for: High-quality permanent draft genome sequence of Rhizobium leguminosarum bv. viciae strain GB30; an effective microsymbiont of Pisum sativum growing in Poland
Source: Stand Genomic Sci. 2015 Jul 16;10:36. doi: 10.1186/s40793-015-0029-6 (PMC4517663; doi:10.1186/s40793-015-0029-6)
Supplement: Additional file 1: Table S1. — Associated MIGS record. [file 40793_2015_29_MOESM1_ESM.doc]

***Table S1.*** *Associated MIGS record.*

| **MIGS-ID** | **Field name** | **Description** |
| --- | --- | --- |
| MIGS-1 | Submit to INSDC/Trace archives |  |
| MIGS-1.1 | PID |  |
| MIGS-1.2 | Trace Archive |  |
| MIGS-2 | MIGS CHECK LIST TYPE |  |
| MIGS-3 | Project Name | GEBA - Root Nodulating Bacteria |
| MIGS-4 | Geographic Location | Poland (Janow near Lublin) |
| MIGS-4.1 | Latitude | 51.387638 |
| MIGS-4.2 | Longitude | 22.369194 |
| MIGS-4.3 | Depth |  |
| MIGS-4.4 | Altitude |  |
| MIGS-5 | Time of Sample collection |  |
| MIGS-6 | Habitat (EnvO) | Host, plant root, root nodule, soil |
| MIGS-6.1 | Temperature | 28 |
| MIGS-6.2 | pH | 5-8 |
| MIGS-6.3 | Salinity |  |
| MIGS-6.4 | Chlorophyll |  |
| MIGS-6.5 | Conductivity |  |
| MIGS-6.6 | Light intensity |  |
| MIGS-6.7 | Dissolved organic carbon (DOC) |  |
| MIGS-6.8 | Current |  |
| MIGS-6.9 | Atmospheric data |  |
| MIGS-6.10 | Density |  |
| MIGS-6.11 | Alkalinity |  |
| MIGS-6.12 | Dissolved oxygen |  |
| MIGS-6.13 | Particulate organic carbon (POC) |  |
| MIGS-6.14 | Phosphate |  |
| MIGS-6.15 | Nitrate |  |
| MIGS-6.16 | Sulfates |  |
| MIGS-6.17 | Sulfides |  |
| MIGS-6.18 | Primary production |  |
| MIGS-7 | Subspecific genetic lineage |  |
| MIGS-9 | Number of replicons |  |
| MIGS-10 | Extrachromosomal elements |  |
| MIGS-11 | Estimated Size | 7.5 Mbp |
| MIGS-12 | Reference for biomaterial or Genome report |  |
| MIGS-14 | Known Pathogenicity | Non-pathogen |
| MIGS-15 | Biotic Relationship | Symbiotic |
| MIGS-16 | Specific Host | [*Pisum sativum*](http://www.theplantlist.org/tpl1.1/record/ild-7792) |
| MIGS-17 | Host specificity or range (taxid) |  |
| MIGS-18 | Health status of Host |  |
| MIGS-19 | Trophic Level |  |
| MIGS-22 | Relationship to Oxygen | Aerobe |
| MIGS-23 | Isolation and Growth conditions | TY medium, 28°C, aerobe |
| MIGS-27 | Nucleic acid preparation | CTAB |
| MIGS-28.1 | Library size | 3,891.5Mbp |
| MIGS-28.2 | Number of reads | 25,943,396 |
| MIGS-28.3 | Vector |  |
| MIGS-29 | Sequencing method | Illumina HiSeq 2000 |
|  |  |  |
| MIGS-30 | Assembly | ALLPATHS v. r41043 |
| MIGS-30.1 | Assembly method |  |
| MIGS-30.2 | Estimated error rate |  |
| MIGS-30.3 | Method of calculation |  |
| MIGS-31 | Finishing strategy |  |
| MIGS-31.1 | Status | High-quality permanent draft |
| MIGS-31.2 | Coverage | 121.9X |
| MIGS-31.3 | Contigs | 78 |
| MIGS-32 | Relevant SOPs |  |
| MIGS-33 | Relevant e-resources |  |
